# Prevalência e Fatores Associados à SRAG por COVID-19 em Adultos e Idosos com Doença Cardiovascular Crônica

**DOI:** 10.36660/abc.20200955

**Published:** 2021-06-10

**Authors:** Karina Mary de Paiva, Danúbia Hillesheim, Cassiano Ricardo Rech, Rodrigo Sudatti Delevatti, Rodrigo Vasconi Sáez Brown, Ana Inês Gonzáles, Patricia Haas

**Affiliations:** 1 Universidade Federal de Santa Catarina Florianópolis SC Brasil Universidade Federal de Santa Catarina (UFSC), Florianópolis, SC – Brasil; 2 Centro Universitário Estácio de Santa Catarina São José SC Brasil Centro Universitário Estácio de Santa Catarina, São José, SC – Brasil

**Keywords:** Adulto, Idoso, Doenças Cardiovasculares, COVID-19, Síndrome Respiratória Aguda Grave, SARS-COV2, Epidemiologia, Prevalência, Comorbidades, Hospitalização

## Abstract

**Fundamento:**

A presença de Doença Cardiovascular (DCV) em indivíduos infectados pela COVID-19 pode implicar em um pior prognóstico.

**Objetivo:**

Descrever a prevalência da Síndrome Respiratória Aguda Grave (SRAG) por COVID-19 e analisar os fatores associados a essa condição em adultos e idosos com doença cardiovascular no Brasil até a 30ª Semana Epidemiológica de 2020.

**Métodos:**

Estudo transversal realizado com dados do Sistema de Informação de Vigilância Epidemiológica da Gripe (SIVEP-Gripe), referente às fichas de notificação de SRAG de indivíduos hospitalizados no Brasil, entre a 1a e 30a Semana Epidemiológica de 2020. Foram incluídos adultos e idosos (≥ 18 anos) com DCV. A variável dependente foi a confirmação de SRAG por COVID-19 e foram analisados fatores relacionados a características sociodemográficas, sinais e sintomas e fatores clínicos. Aplicou-se a regressão de Poisson com variância robusta. O nível de significância adotado foi de 5%.

**Resultados:**

Foram analisadas as notificações de 116.343 indivíduos. Destes, 61,9% obtiveram diagnóstico de SRAG por COVID-19. A prevalência do desfecho foi 4% menor nas mulheres (IC95%: 0,94–0,99) e 18% menor em zonas rurais (IC95%: 0,77–0,87). Observou-se prevalência maior na faixa etária de 50 a 59 anos (IC95%: 1,09–1,48) e na região nordeste (IC95%: 1,72–1,91). Febre, tosse, internação em UTI, uso de suporte ventilatório e caso nosocomial também foram significativamente associados a uma maior probabilidade de SRAG por COVID-19 nesses indivíduos.

**Conclusão:**

Há alta prevalência de SRAG por COVID-19 em adultos e idosos com DCV no Brasil. Associaram-se fatores relacionados a características sociodemográficas, clínicas, sinais e sintomas.

## Introdução

A Síndrome Respiratória Aguda Grave (SRAG) é um dos desfechos relacionados à infecção pelo coronavírus, denominada Sars-CoV-2, e tem se configurado em uma pandemia que tem gerado implicações sociais, financeiras e psicológicas em todo o mundo.^1^ A doença foi caracterizada como uma pandemia, com 15.581.009 casos confirmados e 635.173 mortes em todo o mundo até 23 de agosto de 2020.^2^

A presença de Doença Cardiovascular (DCV) em indivíduos infectados pela COVID-19 pode implicar em um pior prognóstico, além de estar associada a uma maior taxa de letalidade.^3^ Dados sugerem que a lesão cardíaca aguda, o choque cardiogênico e a arritmia cardíaca estavam presentes, respectivamente, em 7,2, 8,7 e 16,7% dos pacientes após infecção pela COVID-19, e destacam que a estadia em Unidade de Terapia Intensiva (UTI) pode aumentar essa prevalência.^4^

As DCV se destacam como um importante problema de saúde pública nos países de baixa e média renda, tendo em vista o aumento da carga dessas doenças, seja com relação às comorbidades em função do convívio com as mesmas, seja pelos gastos públicos envolvidos.^5^ De acordo com dados da Pesquisa Nacional de Saúde (PNS), a prevalência de DCV entre brasileiros adultos com idade maior ou igual a 18 anos era de 4,2%, apresentando gradiente crescente com o aumento da idade, destacando-se a prevalência de 11,4% nos idosos.^6^

Estudos envolvendo dados nacionais em um momento de pandemia auxiliam no entendimento e no direcionamento de ações mais efetivas e no planejamento a longo prazo. As ações de isolamento social adotadas para o enfrentamento da COVID-19 são distintas entre as macrorregiões brasileiras. Destacam-se as preocupações quanto a morbidades pré-existentes, como é o caso das DCV; os pacientes são orientados a seguir restrições de atividades que impõem limitações que poderão comprometer o controle das complicações advindas do convívio com as DCV, além do acompanhamento médico restrito nesse período.

Diante desse contexto, o objetivo deste estudo foi descrever a prevalência de SRAG por COVID-19 e analisar os fatores associados a essa condição em adultos e idosos com DCV crônica no Brasil até a 30ª Semana Epidemiológica de 2020.

## Métodos

### Delineamento e fonte de dados

Trata-se de um estudo transversal realizado com dados do Sistema de Informação de Vigilância Epidemiológica da Gripe (SIVEP-Gripe), disponibilizados pela plataforma *opendatasus* , disponível em https://opendatasus.saude.gov.br/. O Ministério da Saúde (MS), por meio da Secretaria de Vigilância em Saúde (SVS), desenvolve a vigilância da SRAG no Brasil desde 2009. Em 2020, a COVID-19 foi incorporada à rede de vigilância do Influenza e outros vírus respiratórios. Neste estudo, utilizaram-se dados das fichas de notificação de SRAG de indivíduos hospitalizados.

Os casos de SRAG são definidos por indivíduos que atendam os seguintes critérios: (a) febre, mesmo que autorreferida; (b) tosse ou dor de garganta; (c) dispneia ou saturação de O_2_ < 95% ou desconforto respiratório; e (d) que tenham sido hospitalizados ou evoluído a óbito independentemente de hospitalização prévia.^7^

Foram incluídos neste estudo adultos e idosos (≥ 18 anos) com DCV hospitalizados com SRAG. Os indivíduos deveriam apresentar diagnóstico completo do caso na ficha de notificação (SRAG ou SRAG por COVID-19). O período de análise ocorreu até a 30^a^ Semana Epidemiológica de 2020.^8^

### Variável dependente

A variável dependente foi a confirmação de SRAG por COVID-19 (não; sim). A variável “classificação final do caso”, presente no banco de dados, apresentava as seguintes categorias de resposta: SRAG por influenza; SRAG por outros vírus respiratórios; SRAG por outro agente etiológico; SRAG não especificado; e COVID-19. Dessa forma, as categorias de SRAG foram agrupadas e a variável dependente foi categorizada em “outros tipos de SRAG” e “SRAG por COVID-19”. Dentre os casos analisados, 95,9% foram diagnosticados laboratorialmente, 0,4% por meio de vínculo epidemiológico e 3,7% de forma clínica.

### Variáveis independentes

As variáveis foram analisadas em três blocos distintos: fatores sociodemográficos, sinais e sintomas, e fatores clínicos. Os fatores sociodemográficos compreenderam: sexo (masculino; feminino), faixa etária (18 a 29; 30 a 39; 40 a 49; 50 a 59; 60 a 69; 70 a 79; 80 ou mais), raça (branca; preta; amarela; parda; indígena), macrorregião de residência (Sul; Sudeste; Centro-Oeste; Nordeste; Norte) e zona de residência do paciente (urbana; rural; periurbana). No bloco de sinais e sintomas, foram incluídos: febre (não; sim), tosse (não; sim), dispneia (não; sim) e saturação de O2 <95% (não; sim). Com relação aos fatores clínicos, analisou-se: internação em UTI (não; sim), uso de suporte ventilatório (não; sim, invasivo; sim, não invasivo) e caso nosocomial, ou seja, caso de SRAG com infecção adquirida após internação (não; sim).

### Análise de dados

Foi realizada uma análise descritiva de todas as variáveis por meio do cálculo das frequências relativas. Para a identificação dos fatores associados à confirmação de SRAG por COVID-19, inicialmente, foram estimadas as prevalências do desfecho segundo as variáveis da pesquisa, através do teste de X^2^ de Pearson, com nível de significância de 5%. Posteriormente, aplicou-se o modelo de Poisson com variância robusta, tanto bivariado quanto multivariado. Foram estimadas as razões de prevalência (RP) bruta e ajustada dos dados, com seus respectivos intervalos de confiança de 95% (IC 95%). Utilizou-se a RP como medida de associação por se mostrar mais conservadora perante altas prevalências de desfechos.^9^

Para a entrada das variáveis na análise multivariada, considerou-se valor de *p* menor que 0,20 na análise bivariada. As variáveis foram introduzidas de uma só vez (método direto de seleção de variáveis). No modelo final, foram consideradas associadas as variáveis com valor de p ≤ 0,05. As análises foram efetuadas no *software Stata* , versão 14.0 (https://www.stata.com).

### Aspectos Éticos

Por tratarem-se de dados secundários, de domínio público e sem a identificação dos participantes, dispensou-se a aprovação do Comitê de Ética em Pesquisa com Seres Humanos (CEPSH), conforme resolução nº 510, de 7 de abril de 2016, do Conselho Nacional de Saúde (CNS).^10^

## Resultados

As notificações de 116.343 pacientes com DCV foram analisadas neste estudo. Desses, 61,9% foram diagnosticados com SRAG por COVID-19. Com relação à caracterização da amostra, a maioria era do sexo masculino (52,8%), de raça branca (51,3%), da macrorregião sudeste (58,3%) e residia em zonas urbanas (95,7%). Além disso, foi observada maior prevalência de indivíduos com 60 anos ou mais de idade (73,6%). Com relação à análise bivariada, todas as variáveis foram associadas à maior prevalência de SRAG por COVID-19, exceto a faixa etária de 80 anos ou mais e indivíduos que residiam em zonas periurbanas ( Tabela 1 ).

**Tabela 1:** – Caracterização e análise bivariada dos fatores sociodemográficos associados a confirmação de SRAG por COVID-19 em adultos e idosos com doença cardiovascular crônica. Brasil, 2020. (N=116.343)

Variável	Percentual da amostra total %	Prevalência de SRAG por COVID-19 %	p*	RP bruta (IC95%)
**Sexo**			<0,001	
Masculino	52,8	63,8		1,00
Feminino	47,2	59,8		0,93 (0,92-0,95)
**Faixa etária**			<0,001	
18 a 29	0,7	53,3		1,00
30 a 39	2,8	63,6		1,19 (1,07-1,32)
40 a 49	7,6	67,3		1,26 (1,14-1,39)
50 a 59	15,3	67,4		1,26 (1,14-1,39)
60 a 69	23,4	65,2		1,22 (1,11-1,34)
70 a 79	25,0	61,1		1,14 (1,04-1,26)
80 ou mais	25,2	54,9		1,02 (0,93-1,13)
**Raça**			<0,001	
Branca	51,3	55,0		1,00
Preta	7,7	60,8		1,10 (1,06-1,14)
Amarela	1,6	64,0		1,16 (1,08-1,24)
Parda	39,2	65,1		1,18 (1,16-1,20)
Indígena	0,2	73,3		1,33 (1,12-1,58)
**Macrorregião**			<0,001	
Sul	10,9	39,9		1,00
Sudeste	58,3	61,3		1,53 (1,49-1,58)
Centro-Oeste	5,6	61,8		1,54 (1,48-1,61)
Nordeste	18,8	73,1		1,82 (1,77-1,88)
Norte	6,8	72,0		1,80 (1,73-1,87)
**Zona de residência**			<0,001	
Urbana	95,7	61,7		1,00
Rural	3,9	53,1		0,86 (0,82-0,89)
Periurbana	0,4	57,8		0,93 (0,81-1,07)

*Teste qui-quadrado de Pearson; valor de p <0,05; IC95%: intervalo de 95% de confiança.

No que se refere aos sinais e sintomas, a maioria apresentou febre (69,5%), tosse (79,3%), dispneia (82,8%) e saturação de O_2_ <95% (74,0%). Sobre os fatores clínicos, a maioria não necessitou ser internada em UTI (59,9%) e não se tratou de caso nosocomial (96,7%). Contudo, 50% dos indivíduos necessitou suporte ventilatório invasivo. Os sinais e sintomas associados à maior prevalência de SRAG por COVID-19 foram: febre, tosse e saturação de O_2_ <95% ( *p* <0,05). Com relação aos fatores clínicos, associou-se ao desfecho a internação em UTI, o uso de suporte ventilatório e caso nosocomial ( Tabela 2 ).

**Tabela 2:** – Caracterização e análise bivariada dos sinais e sintomas e fatores clínicos associados a confirmação de SRAG por COVID-19 em adultos e idosos com doença cardiovascular crônica. Brasil, 2020. (N=116.343)

Variável	Percentual da amostra total %	Prevalência de SRAG por COVID-19 %	p*	RP bruta (IC95%)
**Febre**			<0,001	
Não	30,5	49,3		1,00
Sim	69,5	67,4		1,36 (1,34-1,39)
**Tosse**			<0,001	
Não	20,7	52,1		1,00
Sim	79,3	64,2		1,23 (1,20-1,25)
**Dispneia**			0,271	
Não	17,2	61,6		1,00
Sim	82,8	61,2		0,99 (0,97-1,01)
**Saturação 0**_**2**_ **<95%**			<0,001	
Não	26,0	57,4		1,00
Sim	74,0	62,3		1,08 (1,06-1,10)
**Internado em UTI**			<0,001	
Não	59,9	58,6		1,00
Sim	40,1	65,7		1,19 (1,10-1,13)
**Uso de suporte ventilatório**			<0,001	
Não	25,7	55,5		1,00
Sim, invasivo	24,3	66,7		1,20 (1,17-1,22)
Sim, não invasivo	50,0	61,7		1,12 (1,09-1,13)
**Caso nosocomial**			<0,001	
Não	96,7	60,0		1,00
Sim	3,3	66,4		1,10 (1,05-1,15)

*Teste qui-quadrado de Pearson; Em negrito valor de p <0,05; UTI: Unidade de Terapia Intensiva; IC95%: intervalo de 95% de confiança.

No modelo final ajustado, a prevalência de SRAG por COVID-19 foi 4% menor nas mulheres, quando comparadas aos homens (RP=0,96; IC 95%: 0,94–0,99) e 18% menor em indivíduos que residiam em zonas rurais (RP=0,82; IC 95%: 0,77–0,87), quando comparados a indivíduos que residiam em zonas urbanas. Por outro lado, destaca-se a prevalência 1,27 vezes maior na faixa etária de 50 a 59 anos (IC 95%: 1,09–1,48), e 1,81 vezes maior na região nordeste (IC 95%: 1,72–1,91). Febre (RP=1,24; IC 95%: 1,20–1,27), tosse (RP=1,12; IC 95%: 1,09–1,16), internação em UTI (RP=1,08; IC 95%: 1,05–1,11), uso de suporte ventilatório invasivo (RP=1,14; IC 95%: 1,09–1,19), uso de suporte ventilatório não invasivo (RP=1,11; IC 95%: 1,07–1,14) e caso nosocomial (RP=1,12; IC 95%: 1,05–1,20) foram estatisticamente associados a uma maior probabilidade de SRAG por COVID-19 ( Tabela 3 ).

**Tabela 3:** – Análise multivariada avaliando os fatores sociodemográficos, sinais e sintomas e fatores clínicos associados a confirmação de SRAG por COVID-19 em adultos e idosos com doença cardiovascular crônica. Brasil, 2020

Variável	Modelo final	
	RP Ajustada (IC95%)	Valor de p*
**Sexo**		0,010
Masculino	1,00	
Feminino	0,96 (0,94-0,99)	
**Faixa etária**		
18 a 29	1,00	
30 a 39	1,17 (0,99;1,38)	0,056
40 a 49	1,25 (1,07-1,46)	0,004
50 a 59	1,27 (1,09-1,48)	0,002
60 a 69	1,21 (1,04-1,41)	0,010
70 a 79	1,17 (0,96-1,29)	0,148
80 ou mais	0,99 (0,85-1,16)	0,981
**Macrorregião**		
Sul	1,00	
Sudeste	1,45 (1,39-1,51)	<0,001
Centro-Oeste	1,35 (1,26-1,45)	<0,001
Nordeste	1,81 (1,72-1,91)	<0,001
Norte	1,71 (1,62-1,82)	<0,001
**Zona de residência**		
Urbana	1,00	
Rural	0,82 (0,77-0,87)	<0,001
Periurbana	0,92 (0,76-1,12)	0,451
**Febre**		
Não	1,00	
Sim	1,24 (1,20-1,27)	<0,001
**Tosse**		
Não	1,00	
Sim	1,12 (1,09-1,16)	<0,001
**Internado em UTI**		
Não	1,00	
Sim	1,08 (1,05-1,11)	<0,001
**Uso de suporte ventilatório**		
Não	1,00	
Sim, invasivo	1,14 (1,09-1,19)	<0,001
Sim, não invasivo	1,11 (1,07-1,14)	<0,001
**Caso nosocomial**		
Não	1,00	<0,001
Sim	1,12 (1,05-1,20)	

No modelo final as variáveis foram ajustadas entre si; *Em negrito valor de p <0,05; UTI: Unidade de Terapia Intensiva; IC95%: intervalo de 95% de confiança.

## Discussão

Dentre os adultos e idosos hospitalizados que possuíam DCV, 61,9% obtiveram diagnóstico de SRAG por COVID-19. A prevalência do desfecho foi 4% menor nas mulheres e 18% menor em indivíduos que residiam em zonas rurais. Por outro lado, foi observada maior prevalência na faixa etária de 40 a 69 anos e na região nordeste. Febre, tosse, internação em UTI, uso de suporte ventilatório e caso nosocomial foram significativamente associados a uma maior probabilidade de SRAG por COVID-19.

Doenças crônicas podem ser consideradas fatores de risco à infecção por COVID-19 em função de sua susceptibilidade a maior morbimortalidade associada.^11 , 12^ Sendo assim, indivíduos com DCV prévias podem estar mais vulneráveis a quadros mais graves da infecção,^13^ considerando a fragilidade do sistema de cada indivíduo, oportunizando assim a ação potencial do vírus e corroborando com os dados encontrados nesta pesquisa, cuja prevalência de diagnóstico confirmado para COVID-19 nos pacientes com DCV hospitalizados foi alta.

Mesmo antes da pandemia, as DCV eram comorbidades comuns em diagnósticos de SRAG, sendo que estas podem elevar em doze vezes o risco de mortalidade associada.^14 , 15^ Embora não tenham sido avaliados os óbitos entre os participantes, um estudo desenvolvido por Zhang,^16^ em Wuhan na China, evidenciou que a mortalidade por COVID-19 em pacientes com DCV apresentou prevalência superior (22,2%) em relação à população geral do estudo (9,8%).

Estudos apontam que homens têm maior risco de evoluir para um quadro de maior gravidade da COVID-19,^17^ indicando possível influência de fatores biológicos intrínsecos ao sexo como também de fatores socioculturais e comportamentais. Esses dados parecem estar melhor consolidados em estudos populacionais chineses^18^ e europeus^19 , 20^ nos quais dados desagregados por sexo mostram números absolutos de contaminação semelhantes entre homens e mulheres, porém com pior evolução em homens, principalmente com DCV. Em publicação recente no periódico *Biology of Sex Differences* , dados epidemiológicos de países como Itália, China, Espanha, França, Alemanha e Suíça foram analisados e reforçaram essa hipótese. Esses dados agrupados indicam, ainda, que essa diferença nas taxas de infecção e pior prognóstico entre os sexos pode ser mais pronunciada em indivíduos de meia idade (50 a 59 anos).^17^

Uma das possíveis explicações para menor prevalência de SRAG por COVID-19 em mulheres é a variação entre a resposta imunológica e a susceptibilidade a infecções virais entre os sexos, podendo levar a diferenças na gravidade e na evolução da doença.^17^ Além disso, parece haver diferenças significativas na regulação e expressão de proteínas que participam do processo fisiopatológico do SARS-CoV-2 entre os sexos. Dados como a diferença entre nível circulante, atividade e expressão de enzima conversora de angiotensina 2^21 , 22^ e a serina protease transmembrana tipo 2^23^ corroboram essa teoria. Além disso, um estudo realizado no Brasil apontou que mulheres com e sem doenças crônicas não transmissíveis (DCNT) utilizam mais os serviços de saúde, quando comparadas aos homens.^24^ Esse fato pode ser atribuído à maior percepção quanto aos sinais e sintomas de doenças, maior prevalência de realização de exames e maiores práticas de promoção e prevenção à saúde, contribuindo para melhores desfechos em saúde e menores taxas de infecção.^24^

Apesar de se observar uma prevalência 18% menor de SRAG por COVID-19 em zonas rurais, provavelmente pela baixa densidade populacional, observam-se também incidência e mortalidade elevadas em regiões rurais e remotas, como Amazonas e Amapá, o que pode ser justificado pela dificuldade de acesso a cuidados intensivos.^25 , 26^ Corroborando esses achados, uma análise epidemiológica realizada nos Estado Unidos identificou uma maior taxa de infecção por SARS-CoV-2 na população urbana; entretanto, indivíduos negros, com idades entre 25 e 49 anos, fumantes e obesos estiveram relacionados a taxas de prevalência de COVID-19 aumentadas em áreas rurais.^25^

As maiores prevalências observadas nas macrorregiões norte e nordeste podem se apresentar como um problema de saúde pública em potencial, tendo em vista as desigualdades regionais brasileiras.^27^ Esse quadro pandêmico expõe fragilidades na atenção e assistência à saúde no Brasil e reforça as questões de desigualdade nas regiões norte e nordeste, no que tange à contingência de profissionais, infraestrutura e capacidade para a produção e realização de testes diagnósticos, questões estas anteriores à pandemia e que persistiram no enfrentamento do atual quadro epidemiológico.^28^

Um relatório produzido pela Fundação Oswaldo Cruz (FIOCRUZ) (2020)^29^ buscou classificar indicadores de vulnerabilidade (A-menos vulnerável a E-mais vulnerável) em nível municipal, com o intuito de criar estimativa de risco de propagação da COVID-19 nos estados brasileiros. Para isso, foram considerados fatores como expectativa de vida ao nascer, índice de GINI — que mede desigualdade e distribuição de renda —, componente de escolaridade do índice de desenvolvimento humano (IDHedu), % população vivendo em situação de extrema pobreza, % população vivendo em área urbana, % de pessoas em domicílios com abastecimento de água e esgotamento sanitário inadequados, % de domicílios com água encanada e % de domicílios sem energia elétrica. Os dados mostraram que os municípios da região norte e nordeste foram considerados mais vulneráveis, pertencentes às classes C, D e E, e que as capitais menos populosas, como Teresina, Maceió, Aracajú, Palmas, Rio Branco e Porto Velho apresentaram alto potencial de disseminação.

Neste estudo, observou-se que os sinais e sintomas mais frequentes associados à confirmação de SRAG por COVID-19, foram dispneia, tosse e febre, com associação estatisticamente significante apenas para febre e tosse. A análise de dados de 4.203 pacientes chineses identificou que os sintomas mais comuns associados à infecção por COVID-19 foram febre, tosse e dispneia (80,5, 58,3 e 23,8%); com relação às comorbidades, foram hipertensão, DCV e diabetes (16,4, 12,1 e 9,8%).^30^ Em um estudo retrospectivo desenvolvido por Zhang,^16^ com amostra composta por 380 indivíduos e confirmação para COVID-19, verificou-se que a tosse com produção de escarro foi a condição mais comum em pacientes com DCV quando comparados à população geral.

Em um estudo conduzido por Fang,^12^ foram considerados fatores associados à maior gravidade da doença na população em geral, com maior chance de pior prognóstico, a admissão em UTI (RR: 5,61, IC 95%: 2,68–11,76) e o uso de ventilação invasiva (RR: 6,53, IC 95%: 2,70–15,84). Já o estudo de Wang^4^ mostrou que indivíduos com comorbidades apresentaram a forma mais grave da doença, com maior necessidade de internação em UTI, além da associação encontrada entre o uso de suporte ventilatório e internação em UTI para pacientes com DCV e infecção confirmada para COVID-19, corroborando os achados deste estudo.

Apesar dos esforços a fim de controlar as infecções por COVID-19 adquiridas em ambiente hospitalar, estudos evidenciam que a infecção nosocomial é um agravante no controle da doença.^4 , 30^ No presente estudo, a infecção nosocomial esteve significativamente associada aos casos confirmados de SRAG por COVID-19. Um estudo realizado em Wuhan, na China, epicentro do início da pandemia, demonstrou uma prevalência de infecção nosocomial de 41% de SRAG atribuída à infecção por COVID-19, tendo maior prevalência em relação aos diagnósticos de SRAG em geral.^4 , 30^ Ainda mais preocupante, no estudo de Zhou,^31^ as proporções de infecções nosocomiais entre os pacientes confirmados para COVID-19 nos surtos iniciais da doença foi de 29,3%, reiterando a importância da proteção adequada, especialmente em ambiente hospitalar.

Destaca-se que algumas limitações devem ser consideradas ao interpretar os resultados deste estudo. Foram analisados os dados de adultos e idosos hospitalizados com DCV e, portanto, os resultados não podem ser generalizados para outras populações. Nesse contexto, a falta de variáveis disponíveis representativas de controle das DCV, como medicação utilizada e dados de estilo de vida, limita o ajuste dos achados para o fator *status* /controle das DCV. Ainda, há a influência da qualidade do preenchimento das fichas de notificação e da sua heterogeneidade nas regiões brasileiras, assim como a subnotificação de casos. Além disso, 4,1% dos casos não foram diagnosticados em laboratório. Esse fato pode ser atribuído à escassez de testes diagnósticos e laboratórios certificados para sua realização em algumas regiões do país.^32^ No entanto, foram realizadas outras formas reconhecidas de diagnóstico pelo Ministério da Saúde.^33^

O estudo apresentou seus pontos fortes, destacando-se que a análise de bancos de dados secundários é uma das melhores formas de avaliar a situação epidemiológica de determinada população, sobretudo os bancos de abrangência nacional.

## Conclusão

Conclui-se que há alta prevalência de SRAG por COVID-19 em adultos e idosos com DCV no Brasil. Foram associadas a essa condição fatores relacionados às características sociodemográficas, características clínicas, sinais e sintomas. Por fim, os dados apresentados neste estudo contribuirão para o enfrentamento dessa pandemia ao apresentar achados provenientes de dados nacionais. Também poderão destacar importantes agravantes associados à confirmação de COVID-19, havendo a possibilidade da realização de ações de monitoramento no público alvo.
