# Supplementary material for: Prevalência e Fatores Associados à SRAG por COVID-19 em Adultos e Idosos com Doença Cardiovascular Crônica
Source: Arq Bras Cardiol. 2021 Jun 10;117(5):968–75. [Article in Portuguese] doi: 10.36660/abc.20200955 (PMC8682099; doi:10.36660/abc.20200955)
Supplement: Translation

# Translation: Prevalence and Associated Factors of SARS by Covid-19 in Adults and Aged People with Chronic Cardiovascular Disease

**Authors:** Karina Mary de Paiva, Danúbia Hillesheim, Cassiano Ricardo Rech, Rodrigo Sudatti Delevatti, Rodrigo Vasconi Sáez Brown, Ana Inês Gonzáles, Patricia Haas

**Affiliations:** 1 Brazil Universidade Federal de Santa Catarina (UFSC), Florianópolis, SC – Brazil; 2 Brazil Centro Universitário Estácio de Santa Catarina, São José, SC – Brazil

**Keywords:** Adult, Aged, Cardiovascular Diseases, COVID-19, Severe Acute Respiratory Syndrome, Epidemiology, Prevalence, Comorbidity, Hospitalization

## Abstract

**Background:**

The presence of Cardiovascular Disease (CVD) in individuals infected with COVID-19 may imply a worse prognosis.

**Objective:**

To describe the prevalence of Severe Acute Respiratory Syndrome (SARS) by COVID-19 and to analyze the factors associated with this condition in adults and the elderly with cardiovascular disease in Brazil until the 30th Epidemiological Week of 2020.

**Methods:**

Cross-sectional study conducted with data from the Influenza Epidemiological Surveillance Information System (Sistema de Informação de Vigilância Epidemiológica da Gripe – SIVEP-Gripe), referring to the SARS notification forms of hospitalized individuals in Brazil, between the 1st and 30th Epidemiological Week of 2020. Adults and the aged (≥ 18 years old) with CVD. The dependent variable was SRAG confirmation by COVID-19 and factors related to sociodemographic characteristics, signs and symptoms, and clinical factors were analyzed. Poisson regression with robust variance was applied. The level of significance adopted was 5%.

**Results:**

Notifications from 116,343 individuals were analyzed. Of these, 61.9% were diagnosed with SARS by COVID-19. The prevalence of the outcome was 4% lower in women (95%CI: 0.94–0.99) and 18% lower in rural areas (95%CI: 0.77–0.87). There was a higher prevalence in the 50 to 59 age group (95%CI: 1.09–1.48) and in the northeast region (95%CI: 1.72–1.91). Fever, cough, admission to the ICU, use of ventilatory support, and nosocomial cases were also significantly associated with a higher probability of SRAS by COVID-19 in these individuals.

**Conclusion:**

There is a high prevalence of SARS by COVID-19 in adults and aged people with CVD in Brazil. Factors associated with sociodemographic and clinical characteristics, signs, and symptoms were associated.

## Introduction

Severe Acute Respiratory Syndrome (SARS) is one of the outcomes related to coronavirus infection, called Sars-CoV-2, and has been configured in a pandemic that has generated social, financial, and psychological implications worldwide.^1^ The disease was characterized as a pandemic, with 15,581,009 confirmed cases and 635,173 deaths worldwide as of August 23^rd^, 2020.^2^

The presence of Cardiovascular Disease (CVD) in individuals infected with COVID-19 may result in a worse prognosis, in addition to being associated with a higher lethality rate.^3^ Data suggest that acute cardiac injury, cardiogenic shock, and cardiac arrhythmia were present, respectively, in 7.2, 8.7, and 16.7% of patients after infection by COVID-19, and highlight that the stay in the Intensive Care Unit (ICU) can increase this prevalence.^4^

CVD stand out as an important public health problem in low- and middle-income countries, in view of their increased burden, whether in relation to their related comorbidities, or due to the public spending involved.^5^ According to data from the National Health Survey ( *Pesquisa Nacional de Saúde* – PNS), the prevalence of CVD among Brazilian adults aged 18 years old and older was 4.2%, presenting an increasing gradient with the increasing age, highlighting the prevalence of 11.4% among the aged.^6^

Studies involving national data at a time of pandemic help to understand and direct more effective actions and long-term planning. The social isolation actions adopted to face COVID-19 are different among Brazilian macro-regions. The concerns about pre-existing morbidities are highlighted, as is the case of CVD; patients are instructed to follow activity restrictions that impose limitations which may compromise the control of complications resulting from living with CVD, in addition to restricted medical follow-up during this period.

Given this context, the objective of this study was to describe the prevalence of SARS by COVID-19 and to analyze the factors associated with this condition in adults and aged people with chronic CVD in Brazil until 30^th^Epidemiological Week of 2020.

## Methods

### Study design and data source

This is a cross-sectional study carried out using data from the Influenza Epidemiological Surveillance Information System ( *Sistema de Informação de Vigilância Epidemiológica da Gripe* – SIVEP-Gripe), made available through the opendatasus platform, available at https://opendatasus.saude.gov.br/. The Ministry of Health (MH), through the Health Surveillance Secretariat ( *Secretaria de Vigilância em Saúde* – SVS), has been developing SARS surveillance in Brazil since 2009. In 2020, COVID-19 was incorporated into the surveillance network for Influenza and other respiratory viruses. Data from the SARS notification forms of hospitalized individuals were used in this study.

SARS cases are defined by individuals who meet the following criteria: (a) fever, even if self-reported; (b) cough or sore throat; (c) dyspnea or O_2_saturation <95% or respiratory distress; and (d) who have been hospitalized or died, regardless of previous hospitalization.^7^

This study included adults and aged subjects (≥ 18 years old) with CVD hospitalized with SARS. Individuals should present a complete diagnosis of the case on the notification form (SARS or SARS by COVID-19). The analysis period occurred until the 30^th^Epidemiological Week of 2020.^8^

### Dependent variable

The dependent variable was the confirmation of SARS by COVID-19 (no; yes). The variable “final classification of the case”, present in the database, consisted of the following response categories: SARS due to influenza; SARS by other respiratory viruses; SARS by another etiological agent; unspecified SARS; and COVID-19. Thus, the categories of SARS were grouped and the dependent variable was categorized into “other types of SARS” and “SARS by COVID-19”. Among the cases analyzed, 95.9% were diagnosed in the laboratory, 0.4% through epidemiological relation, and 3.7% clinically.

### Independent variables

The variables were analyzed in three different blocks: sociodemographic factors, signs and symptoms, and clinical factors. Sociodemographic factors included: gender (male; female), age group (18 to 29; 30 to 39; 40 to 49; 50 to 59; 60 to 69; 70 to 79; 80 years old or older), race (white; black; yellow; brown; indigenous), macroregion of residence (South; Southeast; Midwest; Northeast; North) and patient’s residence area (urban; rural; peri-urban). Signs and symptoms included: fever (no; yes), cough (no; yes), dyspnea (no; yes), and O_2_saturation <95% (no; yes). Regarding clinical factors, the following were analyzed: ICU admission (no; yes), use of ventilatory support (no; yes, invasive; yes, non-invasive) and nosocomial case, that is, case of SARS with acquired infection after hospitalization (no; yes).

### Data analysis

A descriptive analysis of all variables was performed by calculating the relative frequencies. To identify the factors associated with the confirmation of SARS by COVID-19, the prevalence of the outcome was initially estimated according to the research variables, using Pearson’s X^2^test, with a significance level of 5%. Subsequently, the Poisson model with robust variance, both bivariate and multivariate, was applied. The raw and adjusted prevalence ratios (PR) of the data were estimated, along with their respective 95% confidence intervals (95%CI). PR was used as a measure of association as it was more conservative in view of the high prevalence of outcomes.^9^

For the entry of the variables in the multivariate analysis, a p-value lower than 0.20 was considered in the bivariate analysis. The variables were introduced at once (direct method of variable selection). In the final model, variables with a p-value ≤ 0.05 were considered associated. The analyses were performed using the Stata software, version 14.0 (https://www.stata.com).

### Ethical aspects

As they are secondary data, available in the public domain and without the identification of the participants, the approval of the Ethics Committee in Research with Human Beings ( *Comitê de Ética em Pesquisa com Seres Humanos* – CEPSH) was waived, according to resolution No. 510, of April 7^th^, 2016, of the National Health Service Council (Conselho Nacional de Saúde – CNS).^10^

## Results

Notifications of 116,343 patients with CVD were analyzed in this study. Of these, 61.9% were diagnosed with SARS by COVID-19. Regarding the characterization of the sample, the majority were male (52.8%), white (51.3%), from the southeastern macro-region (58.3%), and lived in urban areas (95.7%). In addition, a higher prevalence of individuals aged 60 years old or older was observed (73.6%). Regarding the bivariate analysis, all variables were associated with a higher prevalence of SARS by COVID-19, except for the age group of 80 years old or older and individuals living in peri-urban areas ( Table 1 ).

**Table 1:** – Characterization and bivariate analysis of sociodemographic factors associated with confirmation of SARS by COVID-19 in adults and the aged with chronic cardiovascular disease. Brazil, 2020. (N=116,343)

Characteristic	Percentage of the total sample %	Prevalence of SARS by COVID-19 %	p*	Raw PR (95%CI)
**Gender**			<0.001	
Male	52.8	63.8		1.00
Female	47.2	59.8		0.93 (0.92-0.95)
**Age range**			<0.001	
18 to 29	0.7	53.3		1.00
30 to 39	2.8	63.6		1.19 (1.07-1.32)
40 to 49	7.6	67.3		1.26 (1.14-1.39)
50 to 59	15.3	67.4		1.26 (1.14-1.39)
60 to 69	23.4	65.2		1.22 (1.11-1.34)
70 to 79	25.0	61.1		1.14 (1.04-1.26)
80 or more	25.2	54.9		1.02 (0.93-1.13)
**Race**			<0.001	
White	51.3	55.0		1.00
Black	7.7	60.8		1.10 (1.06-1.14)
Yellow	1.6	64.0		1.16 (1.08-1.24)
Brow	39.2	65.1		1.18 (1.16-1.20)
Indigenous	0.2	73.3		1.33 (1.12-1.58)
**Macro-region**			<0.001	
South	10.9	39.9		1.00
Southeast	58.3	61.3		1.53 (1.49-1.58)
Mid-West	5.6	61.8		1.54 (1.48-1.61)
Northeast	18.8	73.1		1.82 (1.77-1.88)
North	6.8	72.0		1.80 (1.73-1.87)
**Residence area**			<0.001	
Urban	95.7	61.7		1.00
Rural	3.9	53.1		0.86 (0.82-0.89)
Peri-urban	0.4	57.8		0.93 (0.81-1.07)

*Pearson’s χ^2^test; p-value <0,05; 95%CI: 95% confidence interval.

With regard to signs and symptoms, the majority presented fever (69.5%), cough (79.3%), dyspnea (82.8%), and O_2_saturation <95% (74.0%). Regarding clinical factors, the majority did not need to be admitted to the ICU (59.9%) and it was not a nosocomial case (96.7%). However, 50% of the individuals required invasive ventilatory support. The signs and symptoms associated with the higher prevalence of SARS by COVID-19 were: fever, cough, and O_2_saturation <95% (p<0.05). Regarding clinical factors, the outcome was associated to ICU admission, use of ventilatory support, and nosocomial case ( Table 2 ).

**Table 2:** – Characterization and bivariate analysis of signs and symptoms and clinical factors associated with confirmation of SARS by COVID-19 in adults and the aged with chronic cardiovascular disease. Brazil, 2020. (N=116,343)

Characteristic	Percentage of the total sample %	Prevalence of SARS by COVID-19 %	p*	Raw PR (95%CI)
**Fever**			<0.001	
No	30.5	49.3		1.00
Yes	69.5	67.4		1.36 (1.34-1.39)
**Cough**			<0.001	
No	20.7	52.1		1.00
Yes	79.3	64.2		1.23 (1.20-1.25)
**Dyspnea**			0.271	
No	17.2	61.6		1.00
Yes	82.8	61.2		0.99 (0.97-1.01)
**O**_**2**_ **Saturation<95%**			<0.001	
No	26.0	57.4		1.00
Yes	74.0	62.3		1.08 (1.06-1.10)
**Hospitalized in the ICU**			<0.001	
No	59.9	58.6		1.00
Yes	40.1	65.7		1.19 (1.10-1.13)
**Use of ventilatory support**			<0.001	
No	25.7	55.5		1.00
Yes, invasive	24.3	66.7		1.20 (1.17-1.22)
Yes, non-invasive	50.0	61.7		1.12 (1.09-1.13)
**Nosocomial case**			<0.001	
No	96.7	60.0		1.00
Yes	3.3	66.4		1.10 (1.05-1.15)

*Pearson’s χ^2^test; In bold, p-value <0,05; ICU: Intensive Care Unit; 95%CI: 95% confidence interval.

In the final adjusted model, the prevalence of SARS by COVID-19 was 4% lower in women, when compared to men (PR=0.96; 95%CI: 0.94–0.99) and 18% lower in individuals who lived in rural areas (PR=0.82; 95%CI: 0.77–0.87), when compared to individuals who lived in urban areas. On the other hand, it is noteworthy the prevalence 1.27 times higher in the age group of 50 to 59 years old (95%CI: 1.09–1.48), and 1.81 times higher in the northeast region (95%CI: 1.72–1.91). Fever (PR=1.24; 95%CI: 1.20–1.27), cough (PR=1.12; 95%CI: 1.09–1.16), admission to the ICU (PR=1, 08; 95%CI: 1.05–1.11), use of invasive ventilatory support (PR=1.14; 95%CI: 1.09–1.19), use of non-invasive ventilatory support (PR=1.11; 95%CI: 1.07–1.14), and nosocomial case (PR=1.12; 95%CI: 1.05–1.20) were statistically associated with an increased likelihood of SARS by COVID-19 ( Table 3 ).

**Table 3:** – Multivariate analysis assessing sociodemographic factors, signs and symptoms, and clinical factors associated with confirmation of SARS by COVID-19 in adults and the aged with chronic cardiovascular disease. Brazil, 2020

Characteristics	Final model	
	Adjusted PR (95%CI)	p-value*
**Gender**		0.010
Male	1.00	
Female	0.96 (0.94-0.99)	
**Age range**		
18 to 29	1.00	
30 to 39	1.17 (0.99;1.38)	0.056
40 to 49	1.25 (1.07-1.46)	0.004
50 to 59	1.27 (1.09-1.48)	0.002
60 to 69	1.21 (1.04-1.41)	0.010
70 to 79	1.17 (0.96-1.29)	0.148
80 or more	0.99 (0.85-1.16)	0.981
**Macro-region**		
South	1.00	
Southeast	1.45 (1.39-1.51)	<0.001
Mid-West	1.35 (1.26-1.45)	<0.001
Northeast	1.81 (1.72-1.91)	<0.001
North	1.71 (1.62-1.82)	<0.001
**Residence area**		
Urban	1.00	
Rural	0.82 (0.77-0.87)	<0.001
Peri-urban	0.92 (0.76-1.12)	0.451
**Fever**		
No	1.00	
Yes	1.24 (1.20-1.27)	<0.001
**Cough**		
No	1.00	
Yes	1.12 (1.09-1.16)	<0.001
**Hospitalized in the ICU**		
No	1.00	
Yes	1.08 (1.05-1.11)	<0.001
**Use of ventilatory support**		
No	1.00	
Yes, invasive	1.14 (1.09-1.19)	<0.001
Yes, non-invasive	1.11 (1.07-1.14)	<0.001
**Nosocomial case**		
No	1.00	<0.001
Yes	1.12 (1.05-1.20)	

In the final model, the variables were adjusted to each other; *In bold, p-value <0,05; ICU: Intensive Care Unit; 95%CI: 95% confidence interval.

## Discussion

Among hospitalized adults and aged people with CVD, 61.9% were diagnosed with SARS by COVID-19. The prevalence of the outcome was 4% lower in women and 18% lower in individuals living in rural areas. On the other hand, a higher prevalence was observed in the age group from 40 to 69 years old and in the northeast region. Fever, cough, ICU admission, use of ventilatory support, and nosocomial case were significantly associated with an increased likelihood of SARS by COVID-19.

Chronic diseases can be considered risk factors for COVID-19 infection due to their susceptibility to greater associated morbidity and mortality.^11 , 12^ Thus, individuals with previous CVD may be more vulnerable to more severe infections,^13^ considering the fragility of each individual’s system, thus providing the potential action of the virus and corroborating with the data found in this research, whose prevalence of confirmed diagnosis for COVID -19 in hospitalized CVD patients was high.

Even before the pandemic, CVD were common comorbidities in diagnoses of SARS, which can elevate the risk of associated mortality by twelve fold.^14 , 15^ Although the deaths among the participants were not evaluated, a study developed by Zhang,^16^ in Wuhan, China, showed that mortality from COVID-19 in patients with CVD had a higher prevalence (22.2%) in relation to the general population of the study (9.8%).

Studies indicate that men are at greater risk of evolving to a more severe condition of COVID-19,^17^ indicating a possible influence of biological factors intrinsic to gender as well as socio-cultural and behavioral factors. These data seem to be better consolidated in Chinese^18^ and European^19 , 20^ populational studies, in which data disaggregated by gender show similar absolute numbers of contamination between men and women, but with a worse evolution in men, especially with CVD. In a recent publication in the journal “Biology of Sex Differences”, epidemiological data from countries such as Italy, China, Spain, France, Germany, and Switzerland were analyzed and reinforced this hypothesis. These grouped data also indicate that this difference in infection rates and a worse prognosis between genders may be more pronounced in middle-aged individuals (50 to 59 years old).^17^

One of the possible explanations for the lower prevalence of SARS by COVID-19 in women is the variation between the immune response and the susceptibility to viral infections between the genders, which can lead to differences in the severity and evolution of the disease.^17^ In addition, there seems to be significant differences in the regulation and expression of proteins that participate in the pathophysiological process of SARS-CoV-2 between the genders. Data such as the difference between circulating level, activity, and expression of angiotensin-converting enzyme 2^21 , 22^ and transmembrane serine protease type 2^23^ corroborate this theory. In addition, a study carried out in Brazil pointed out that women with and without noncommunicable diseases (NCD) use health services more when compared to men.^24^ This fact can be attributed to a greater perception of the signs and symptoms of diseases, a higher prevalence of exams, and greater health promotion and prevention practices, contributing to better health outcomes and lower infection rates.^24^

Despite an 18% lower prevalence of SARS by COVID-19 in rural areas, probably due to the low population density, there is also a high incidence and mortality in rural and remote regions, such as Amazonas and Amapá, which can be justified by the difficulty in access to intensive care.^25 , 26^ Corroborating these findings, an epidemiological analysis carried out in the United States identified a higher rate of SARS-CoV-2 infection in the urban population; however, black individuals, aged between 25 and 49 years, smokers, and obese were related to increased prevalence rates of COVID-19 in rural areas.^25^

The highest prevalences observed in the northern and northeastern macro-regions may present themselves as a potential public health problem, provided the Brazilian regional inequalities.^27^ This pandemic situation exposes weaknesses in health care and assistance in Brazil and reinforces the issues of inequality in the north and northeast regions, with regard to the contingency of professionals, infrastructure, and capacity for the production and performance of diagnostic tests, whose issues date prior to the pandemic and persisted in the current epidemiological situation.^28^

A report produced by the Oswaldo Cruz Foundation (FIOCRUZ) (2020)^29^ sought to classify vulnerability indicators (A-less vulnerable to E-most vulnerable) at the municipal level, in order to create an estimate of the risk of COVID-19 spreading in Brazilian states. For this, factors such as life expectancy at birth, GINI index — which measures inequality and income distribution —, education component of the human development index (IDHedu), % of population living in extreme poverty, % of population living in urban areas, % of people in households with inadequate water supply and sewage, % of households with running water, and % of households without electricity. The data showed that the municipalities in the north and northeast regions were considered more vulnerable, belonging to classes C, D, and E, and that the less populous capitals, such as Teresina, Maceió, Aracajú, Palmas, Rio Branco, and Porto Velho had high potential for dissemination.

In this study, it was observed that the most frequent signs and symptoms associated with the confirmation of SARS by COVID-19 were dyspnea, cough, and fever, with a statistically significant association only for fever and cough. Data analysis of 4,203 Chinese patients identified that the most common symptoms associated with COVID-19 infection were fever, cough, and dyspnea (80.5, 58.3, and 23.8%); with regard to comorbidities, they were hypertension, CVD, and diabetes (16.4, 12.1, and 9.8%).^30^ In a retrospective study developed by Zhang,^16^ with a sample consisting of 380 individuals and confirmation for COVID-19, it was found that cough with sputum production was the most common condition in patients with CVD when compared to the general population.

In a study conducted by Fang^12^ , factors associated with a greater severity of the disease in the general population were considered, with a greater chance of a worse prognosis, admission to the ICU (RR: 5.61, 95%CI: 2.68–11.76) and the use of invasive ventilation (RR: 6.53, 95%CI: 2.70^-^15.84). The study by Wang,^4^ on the other hand, showed that individuals with comorbidities had the most severe form of the disease, with greater need for ICU admission, in addition to the association found between the use of ventilatory support and ICU admission for patients with CVD and confirmed infection for COVID -19, corroborating the findings of this study.

Despite efforts to control COVID-19 infections acquired in a hospital environment, studies show that nosocomial infection is an aggravating factor in the control of the disease.^4 , 30^ In the present study, nosocomial infection was significantly associated with confirmed cases of SARS by COVID-19. A study carried out in Wuhan, China, the epicenter of the beginning of the pandemic, showed a prevalence of nosocomial infection of 41% of SARS attributed to COVID-19 infection, having a higher prevalence in relation to diagnoses of SARS in general.^4 , 30^ Even more worrying, in the study by Zhou,^31^ the proportion of nosocomial infections among patients confirmed for COVID-19 in the initial outbreaks of the disease was 29.3%, reiterating the importance of adequate protection, especially in a hospital environment.

It is highlighted that some limitations must be considered when interpreting the results of this study. Data from adults and aged people hospitalized with CVD were analyzed and, therefore, the results cannot be generalized to other populations. In this context, the lack of available variables representative of CVD control, such as medication used and lifestyle data, limits the adjustment of the findings for the CVD status/control factor. Still, there is the influence of the quality of information completion in notification forms and their heterogeneity in the Brazilian regions, as well as the underreporting of cases. In addition, 4.1% of cases were not diagnosed in the laboratory. This fact can be attributed to the lack of diagnostic tests and certified laboratories to perform them in some regions of the country.^32^ However, other forms of diagnosis were recognized by the Ministry of Health.^33^

The study presented its strengths, highlighting that the analysis of secondary databases is one of the best ways to assess the epidemiological situation of a given population, especially banks with national coverage.

## Conclusion

It was concluded that there is a high prevalence of SARS by COVID-19 in adults and aged people with CVD in Brazil. Factors related to sociodemographic characteristics, clinical characteristics, signs, and symptoms were associated with this condition. Finally, the data presented in this study will contribute to facing this pandemic by presenting findings from national data. They will also be able to highlight important aggravating factors associated with the confirmation of COVID-19, with the possibility of carrying out monitoring actions in the target audience.
